# Direct measurement of transcription rates reveals multiple mechanisms for configuration of the *Arabidopsis* ambient temperature response

**DOI:** 10.1186/gb-2014-15-3-r45

**Published:** 2014-03-03

**Authors:** Kate Sidaway-Lee, Maria J Costa, David A Rand, Bärbel Finkenstadt, Steven Penfield

**Affiliations:** 1 Biosciences, College of Life and Environmental Sciences, University of Exeter, Geoffrey Pope Building, Stocker Road, Exeter EX4 4QD, UK; 2 Systems Biology Centre, University of Warwick, Coventry CV4 7AL, UK; 3 Mathematics Institute, University of Warwick, Coventry CV4 7AL, UK; 4 Department of Statistics, University of Warwick, Coventry CV4 7AL, UK

## Abstract

**Background:**

Sensing and responding to ambient temperature is important for controlling growth and development of many organisms, in part by regulating mRNA levels. mRNA abundance can change with temperature, but it is unclear whether this results from changes in transcription or decay rates, and whether passive or active temperature regulation is involved.

**Results:**

Using a base analog labelling method, we directly measured the temperature coefficient, Q_10_, of mRNA synthesis and degradation rates of the *Arabidopsis* transcriptome. We show that for most genes, transcript levels are buffered against passive increases in transcription rates by balancing passive increases in the rate of decay. Strikingly, for temperature-responsive transcripts, increasing temperature raises transcript abundance primarily by promoting faster transcription relative to decay and not vice versa, suggesting a global transcriptional process exists that controls mRNA abundance by temperature. This is partly accounted for by gene body H2A.Z which is associated with low transcription rate Q_10_, but is also influenced by other marks and transcription factor activities.

**Conclusions:**

Our data show that less frequent chromatin states can produce temperature responses simply by virtue of their rarity and the difference between their thermal properties and those of the most common states, and underline the advantages of directly measuring transcription rate changes in dynamic systems, rather than inferring rates from changes in mRNA abundance.

## Background

The mechanism for ambient temperature sensing in plants is unclear. Control of transcript levels is believed to be important in responses to temperature [1-4] but affects of ambient temperature on transcription and mRNA decay rates have not been measured. According to the work of Arrhenius [5] the temperature coefficient (Q_10_) of biochemical reactions is expected to be 2 to 3 at biological temperatures: yet less than 2% of *Arabidopsis thaliana* genes have a two-fold or greater difference in expression level between 17°C and 27°C [6]. The remaining genes either have rates buffered against changing temperatures, or passive increases in transcription rate must be offset by a balanced increase in decay rate, leading to higher turnover but static steady state levels. Despite this fundamental uncertainty, steady state transcriptomic responses to ambient temperature have been used to infer a role for chromatin modifications in temperature signaling [2,7].

4-Thiouracil (4SU) is a non-toxic base analogue that has been shown to be incorporated into mammalian and yeast mRNA during transcription [8-12]. Biotinylation and column separation allow 4SU-labeled RNA to be separated from unlabeled RNA, and transcriptomic analysis using the separated samples can be used to simultaneously calculate mRNA synthesis and decay rates [8]. Here we use 4SU labeling to measure transcription rates and determine the Q_10_ genome-wide of mRNA synthesis and decay rates in *Arabidopsis thaliana*. We show that ambient temperature has large passive effects on both mRNA synthesis and decay rates, and that where temperature controls transcript abundance it does so by regulating transcription relative to decay and not *vice versa*. Our analysis suggests that transcription factor binding sites and epigenetic state combine to create a complex network of temperature responses in plants.

## Results

**Figure 1:**
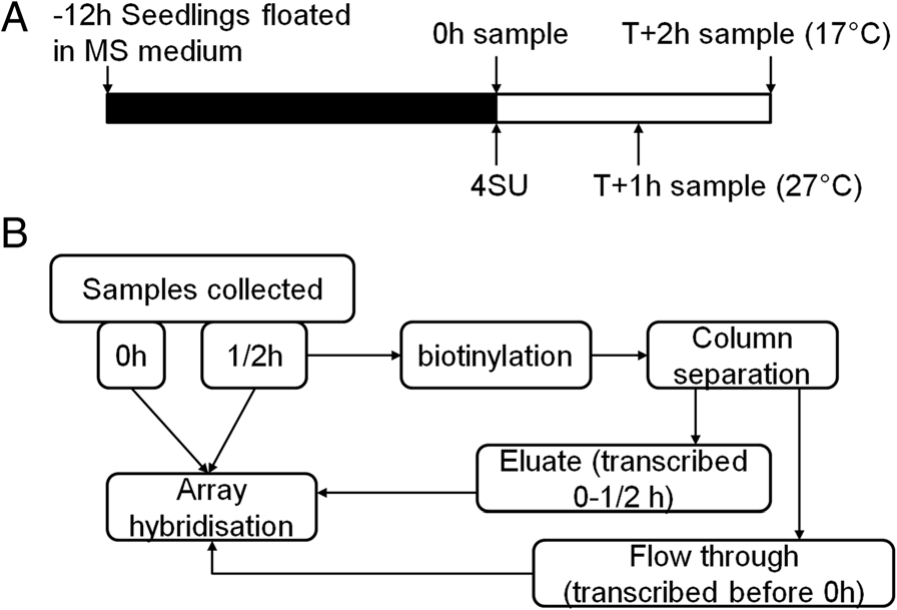
**Schematic of experimental design and workflow. (A)** Experimental design. Seedlings were floated on MS medium 12 hours prior to the experiment, which started on addition of 4SU. RNA was harvested concurrent with 4SU addition, and then either 1 or 2 hours later, depending on the temperature. **(B)** Experimental workflow for harvested samples. Samples at time 0 are extracted and hybridized directly, whereas samples at 1/2 hours are split. Total RNA is hybridized directly and compared to samples at time 0 to give change in steady state level during the labeling period. Total RNA was also split into two fractions by biotinylation and column separation, each of which is also hybridized to give abundance data for transcripts synthesized before and after 4SU addition.

Cells incorporate 4SU into RNA and this has been exploited in mammalian cells [8,11,12] and in yeast [13] to measure mRNA synthesis and decay rates. In order to determine whether plants can take up 4SU we floated intact seedlings in MS medium and monitored 4SU incorporation into RNA by biotinylation and dot blot (Figure S1a in Additional file 1). This clearly showed that plants incorporate 4SU from the environment into RNA and that concentrations as low as 1 mM lead to a signal detectable above background within 1 hour (Figure 1B). The resulting RNA could be separated from unlabeled RNA by biotinylation and passage through a streptavidin column as described previously. At 1.5 mM the flow-through can be depleted of detectable 4SU-labeled RNA, whilst labeled plant RNA is highly concentrated in the fraction recovered from the column [8,13] (Figure S1c in Additional file 1). To maximize recovery we chose a low concentration of 4SU at 1.5 mM [8] as high labeling frequencies are known to lead to binding of fewer more frequently labeled transcripts to the columns and reduce recovery. At this concentration *Arabidopsis* plants treated with 4SU showed the same growth and survival as control plants (Figure S2a in Additional file 1), suggesting 4SU has low toxicity in plants, as in other organisms. Therefore, 4SU dynamics in *Arabidopsis* seedlings resemble those described for other experimental systems. Preliminary experiments showed that RNA turnover was faster at 27°C compared to 12°C (Figure S2b in Additional file 1), suggesting that temperature generally affected transcription rates.

We designed an experiment to determine the Q_10_ of mRNA synthesis and decay rates genome-wide. mRNA abundances were analyzed at two time-points at two temperatures 10°C apart, by microarray (Figure 1). At dawn total RNA was collected from seedlings floating on MS medium at 17°C and at 27°C, and 4SU was added to the remaining samples. After 1 hour at 27°C or 2 hours at 17°C material was flash-frozen, and RNA from this latter collection was separated into 4SU-labeled and unlabeled fractions (Figure S1c in Additional file 1). Total RNA from both the beginning and end of the labeling period, as well as labeled and unlabeled fractions at the end of the labeling period, were analyzed by Affymetrix ATh1 Genechips. Using linear regression [8] the relative contributions of the 4SU-labeled RNA (newly synthesized) fractions and unlabeled RNA (pre-existing, was transcribed before 4SU addition) to the total RNA fraction could be calculated, and the expression level of each gene normalized accordingly [8] (Figure S3 in Additional file 1; Materials and methods). Combining this information with the change (if any) in steady state levels of each transcript over time, from 12,997 genes called present by MAS5 we could calculate transcription and mRNA decay rates for 7,291 genes expressed in whole seedlings at both temperatures [8] (Materials and methods): dropouts included genes with highly variable expression (often low expressed) at one or both temperatures, or genes with long half lives for which our labeling period was too short to resolve. However, short labeling times are desirable in plants where transcriptional regulation can be highly temporally dynamic even in constant conditions.

**Figure 2:**
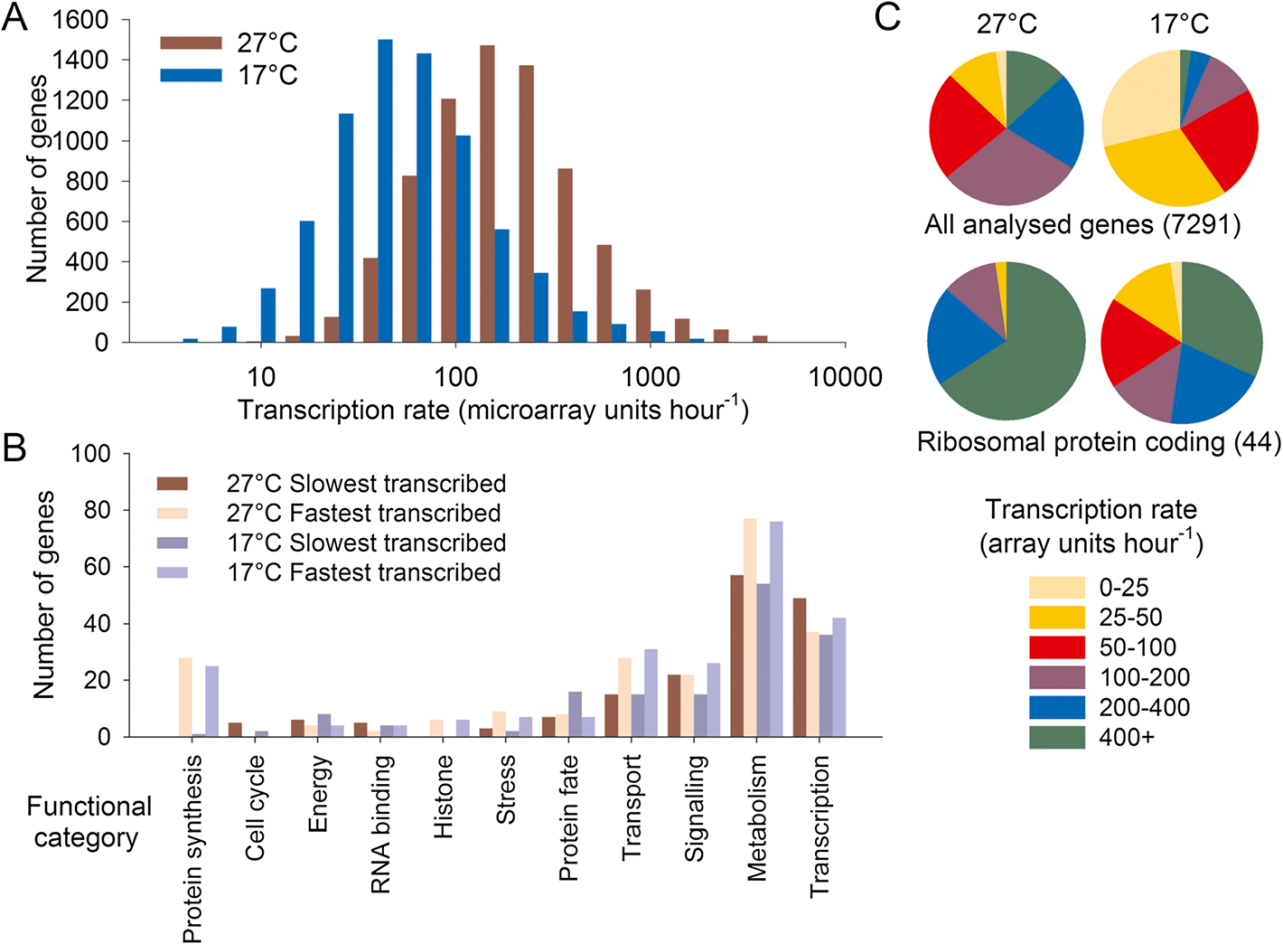
**Genome-wide distribution of transcription rates at 27°C and 17°C. (A)** Histogram to show the distribution of transcription rates, in microarray units per hour, for 7,291 genes for which half-lives and transcription rates could be calculated. **(B)** Functional categories based on Gene Ontology terms of the 5% of genes with fastest and slowest transcription rates, at both temperatures. **(C)** Pie charts to show the distribution of transcription rates for all genes analyzed, at 17°C and 27°C compared to those encoding ribosomal proteins. Data points are calculated from the mean values for each locus from three biological replicate experiments.

Plant transcription rates showed a log normal distribution at both temperatures (Figure 2A; Additional file 2). The wide range of measured rates is good evidence that 4SU incorporation into mRNA depended on variable transcription rates, rather than a single rate of uptake. Gene Ontology (GO) categories, including protein synthesis, metabolism, histones and transport, showed consistently high transcription rates compared to the whole genome, suggesting that genes with house-keeping functions have high transcription rates (Figure 2B,C). Categories with low rates included cell cycle gene expression, which presumably reflects the relative lack of cell division in mature plant tissues. The distribution of transcription rates shifted with temperature, showing that temperature has a major affect on absolute transcription rates in plants (Figure 2A).

**Figure 3:**
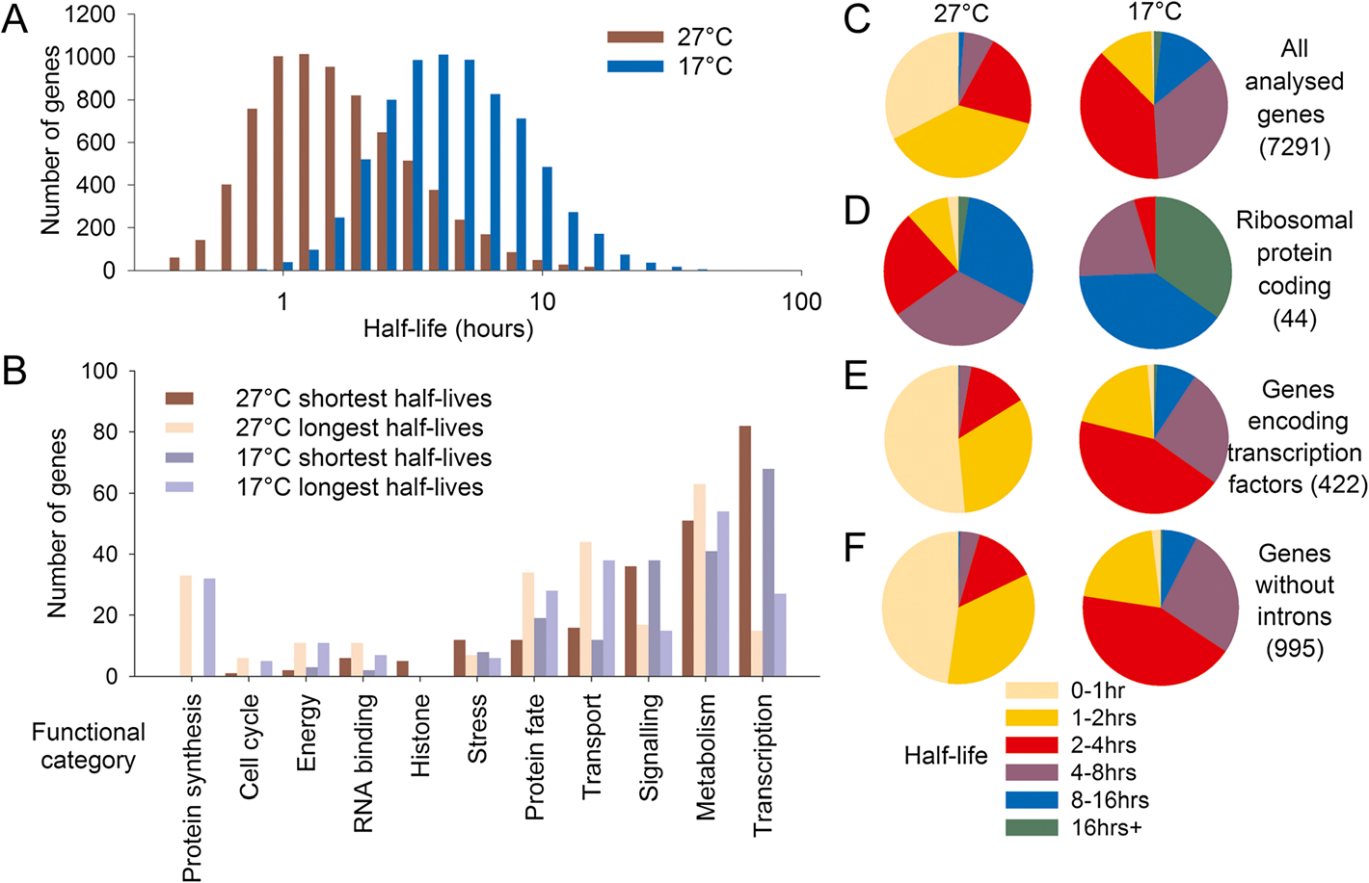
**Genome-wide distribution of mRNA decay rates at 27°C and 17°C. (A)** Histogram to show the distribution of half-lives for 7,291 genes for which half-lives and transcription rates could be calculated. **(B)** Functional categories, based on GO terms, of the 5% of genes with fastest and slowest decay rates (shortest and longest half-lives), at both temperatures. **(C)** Pie charts to show the distribution of half-lives for all 7,291 genes, at 17°C and 27°C. **(D)** Pie charts to show the distributions of half-lives for genes encoding ribosomal proteins. **(E)** Pie charts to show the distributions of half-lives for genes annotated as encoding transcription factors. **(F)** Pie charts to show the distributions of half-lives for intron-less genes, at both temperatures. Data points are calculated from the mean values for each locus from three biological replicate determinations.

The calculated half-lives show wide variation between different individual genes (Figure 3A,C; Additional file 2). At 27°C the mean half life for the genes analyzed is 1.87 hours and this increases to 4.96 hours at 17°C. GO term-based analysis showed that gene categories enriched in genes with slow decay rates encode proteins with housekeeping functions, including basic metabolism and protein synthesis (Figure 3B,D), in agreement with previous analyses [14,15]. Genes with fast decay rates include those involved in signaling, particularly transcription factors, with 50% showing half-lives under 1 hour at 27°C and under 3 hours at 17°C (Figure 3e). Our transcription and decay rate data could be confirmed by RT-PCR using longer labeling time series, showing that rate determination by microarray produces reliable data that can be independently corroborated, despite only measuring values at two time-points (Figure S4 in Additional file 1).

We found no correlation between decay or synthesis rates and mRNA length, UTR length, uracil content, or GC content (Figure S5 in Additional file 1). This is confirmation that the labeling is sufficient and not biasing recovery of newly synthesized RNAs towards those with higher uracil content. However, we found that genes without introns have shorter half-lives (Figure 3F), corroborating similar evidence from a study using transcriptional inhibitors [15]. No relationship between rates of decay and transcription was observed and fold-change in transcript level during labeling correlated with decay rate but not with transcription rate (Figure S5 in Additional file 1).

**Figure 4:**
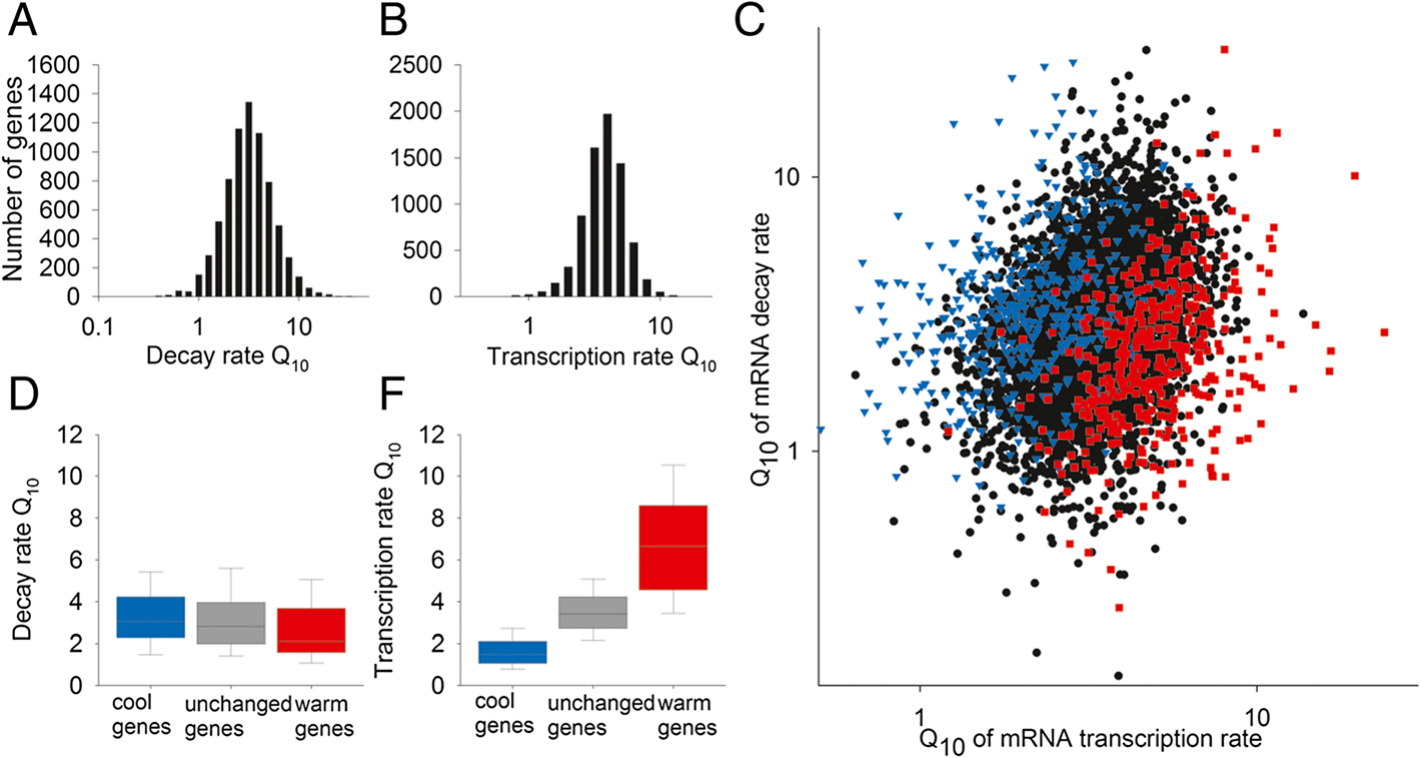
**Increasing temperature affects transcript abundance by active promotion of transcription rates. (A)** Histogram to show the Q_10_ of decay rates at 17°C for all 7,291 genes analyzed; the mean Q_10_ for decay rate is 3.29 and the median 2.81. **(B)** Histogram to show the Q_10_ of transcription rates at 17°C for all 7,291 genes analyzed; the mean Q_10_ for transcription rate is 3.57 and the median 3.40. A number greater than 1 indicates higher transcription/decay rates at 27°C than at 17°C. **(C)** Scatter plot showing the Q_10_ of transcription rate plotted against Q_10_ of decay rate for genes whose transcript abundances are at least 1.5-fold increased (red), 1.5 fold-decreased (blue) or unchanged (black) with increasing temperature. **(D)** Box plot to show the Q_10_ of decay rates for genes with transcript abundances that are higher, lower or unchanged at 27°C compared to 17°C. Boxes show the median and the 5th, 25th, 75th and 95th per percentiles for each group of Q_10_s. **(E)** Box plot to show the Q_10_ of transcription rates for genes with transcript abundances that are higher, lower or unchanged at 27°C compared to 17°C. Boxes show the median and the 5th, 25th, 75th and 95th per percentiles for each group of Q_10_s.

A key question is whether changes in transcript abundance with changes in ambient temperature are related to passive temperature affects or represent the result of an active temperature signaling process. Average decay and transcription rates are both faster at 27°C than at 17°C. At 27°C the mean transcription rate for all genes analyzed is 236.1 microarray units per hour; at 17°C it is 73.0. Likewise, the mean half-life at 27°C is 1.87 hours and at 17°C it is 4.96 hours: this demonstrates that both rates are over three times faster at 27°C compared to 17°C (Figures 2A, 3A and 4). Thus, there is no general mechanism in plants that buffers mRNA abundance against temperature changes, and increasing temperature therefore increases mRNA turnover by affecting both synthesis and decay rates. We calculated the temperature coefficients (Q_10_) for all 7,292 genes for both transcription and decay rates (Figure 4A, B). These were found to follow a log-normal distribution, indicating that the mean Q_10_ is influenced by some genes with particularly large Q_10_. The mean Q_10_ at 17°C for synthesis rates for all genes analyzed is 3.57 and the median 3.40. For decay rates the mean Q_10_ at 17°C is 3.29 and the median 2.80. Both means are higher than the Q_10_ of two- to three-fold predicted by the Arrhenius equation for a passive increase in rates, suggesting the presence of active response processes, as well as passive changes.

Because splicing has also previously been shown to play a role in the control of gene expression by temperature [16], we examined the affect of intron number on the distribution of synthesis and decay rate temperature coefficients (Figure S6 in Additional file 1). We found no evidence that intron presence or number could influence Q_10_ of either transcript synthesis or decay on a global scale, suggesting that temperature affects a limited number of Q_10_s in this way, or that temperature-regulated splicing does not frequently affect abundance of all detectable RNA species from a locus.

Ambient temperature has been shown to modify the transcript levels of approximately 2% of the *Arabidopsis* genome [2,6]. Increasing transcript levels at higher temperatures could be achieved by a faster transcription rate or a slower decay rate. Our dataset includes 417 genes whose transcript abundances are more than 1.5-fold and significantly higher at the higher temperature, and 458 genes with transcript abundances that are more than 1.5-fold and significantly lower at the higher temperature. These two sets of genes are clearly separated by Q_10_ for transcription rate, but not decay rate Q_10_s (Figure 4C). Therefore, a Q_10_ of transcription that diverges from the mean is associated with mRNA species that change in abundance with temperature. Importantly, most transcripts that are more abundant at 27°C still show increases in decay rates with increasing temperature (Figure 4C), and increases in transcript abundance occur because transcription rate Q_10_s exceed decay rate Q_10_s.

We divided our dataset into three groups: genes whose levels rise at least two-fold with increasing temperature (warm genes (WGs)), genes whose levels stay roughly constant (unchanged genes (UGs)) and genes whose levels rise at least two-fold with decreasing temperatures (cool genes (CGs)). We then calculated the Q_10_ of transcription and decay for each group (Figure 4D, E). Transcription rate Q_10_s of the WGs are significantly and remarkably higher than those of the UGs, which in turn are higher than those of the CGs (Figure 4E). In contrast, only small changes in decay rate Q_10_s were noted between the three groups and these were dwarfed by those observed for transcription rates. Together, our data shows that large changes in transcription rate are important in determining transcript abundance changes with ambient temperature, and that, on average, this is achieved by overcoming smaller, predominantly passive changes in decay rate.

**Figure 5:**
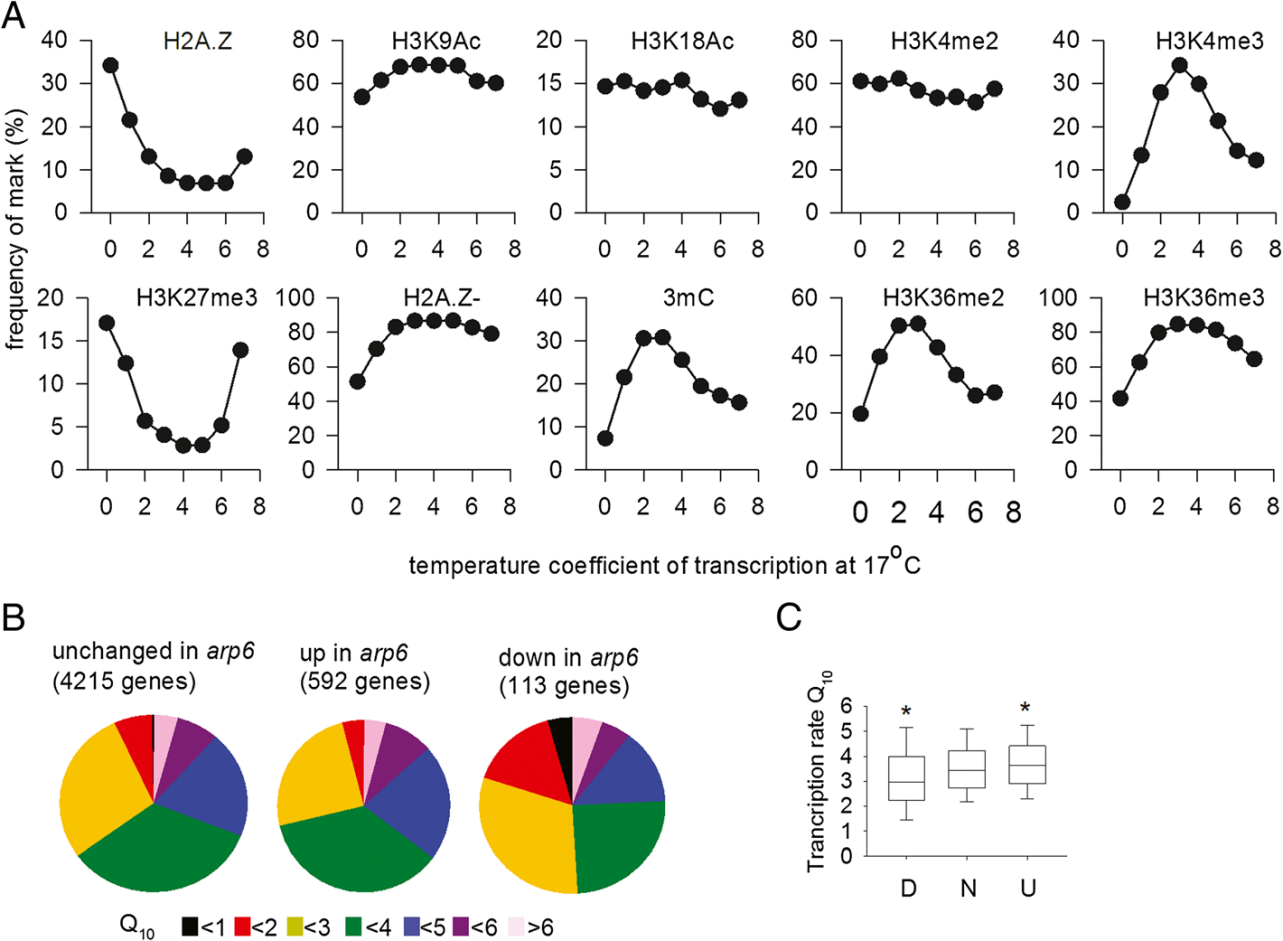
**Epigenetic control of transcription rate Q**_**10 **_**in *Arabidopsis*. (A)** The frequency distribution of chromatin marks among genes with differing temperature coefficients of transcription. Data (from Coleman-Derr *et al*. [20] and Luo *et al*. [21]) show the percentage of genes in each Q_10_ category with each epigenetic character. Numbers of genes in each Q_10_ group are 0 < Q_10_ < 1, 41; 1 < Q_10_ < 2, 527; 2 < Q_10_ < 3, 2,039; 3 < Q_10_ < 4, 2,447; 4 < Q_10_ < 5, 1,389; 5 < Q_10_ < 6, 526; 6 < Q_10_ < 7, 174; 7 < Q_10_ < 8, 82. **(B)** Pie charts showing the distribution of Q_10_s of transcription rates for genes increased, decreased or unchanged in *arp6* loss of function lines, using data from Kumar and Wigge [2]. **(C)** Box plot to show median, 50% of data and 95% of data distributions for transcription rate Q_10_s for ARP6 down-regulated genes (D), up-regulated genes (U) and genes with ARP6-independent expression (N). Asterisks show that the transcription rate Q_10_s for genes with increased or decreased abundance in *arp6* are significantly different from the genes with ARP6-independent expression (*P* = 0.0003 for genes decreased in *arp6* and for increased *P* = 0.0001).

Because we can clearly show that regulation of transcription but not decay rates is central to the ambient temperature regulation of gene expression, we explored the possibility that key transcription factors or epigenetic states play a role in this process. For instance, previously it has been suggested that ARP6 and the SWR1 complex control ambient temperature responses by functioning in the replacement of histone H2A with H2A.Z at lower temperatures [2]. The relationship between other epigenetic states and temperature responsiveness has not been clearly addressed. In addition, signaling pathways controlled by temperature-regulated transcription factors have been shown to play a role in temperature signaling, including heat shock factors, basic helix-loop-helix (bHLH) transcription factors, FLOWERING LOCUS C (FLC), ABA response element (ABRE)-binding factors, and circadian clock genes [1,4,17-19]. Because our data showed that control of transcription rate is important in RNA abundance responses to ambient temperature, we measured the frequency of each feature in gene groups with Q_10_s ranging from <1 to <8, based on published *Arabidopsis* seedling chromatin immunoprecipitation data at 22°C [20,21], or the presence of an implicated transcription factor binding site up to 1 kb upstream of the transcription start site using The Arabidopsis Information Resource (TAIR) 10 annotation (Figure 5A).

This analysis revealed some striking findings (Figure 5A). High levels of gene body H2A.Z [20] were more frequent in genes with Q_10_s lower than 2, with frequency rising from 8% of genes with average Q_10_s to 35% of genes with Q_10_s lower than 1. This supports previous data suggesting that H2A.Z has a role in ambient temperature-regulation of transcription [2], but suggests that gene body H2A.Z rather than presence at the transcription start site is important for differential expression in response to temperature changes. In contrast, genes that are depleted in gene body H2A.Z showed little bias in Q_10_. Analysis of the Q_10_ of genes whose expression is altered in *arp6* mutants at 12°C [2] found that genes with higher than average transcription rate Q_10_s were unaffected in abundance in *arp6* mutants at 12°C, whereas genes with Q_10_s below the mean were lower in abundance in *arp6* mutants compared to the wild type. This further supports the hypothesis that between 12°C and 27°C H2A.Z/ARP6 activity is associated with low transcription rate Q_10_. For other marks, H3K27me3 was frequently associated with genes with both high and low Q_10_s and was depleted in the gene bodies of passively regulated genes. In contrast, other more common marks, including DNA methylation, H3K4me3, H3K36me3 and H3k36me2, were preferentially present in genes with average Q_10_s. Given that the majority of genes have near-average Q_10_s, the high frequency of these states combined with common properties determines the mean temperature response of transcription (see Discussion for details). Together our data show that H2A.Z and H3K27me3 play a role in the generation of more thermostable transcription rates (that is, with a low Q_10_), but that H3K27me3 repression is also more frequently associated with greater than average Q_10_s.

**Figure 6:**
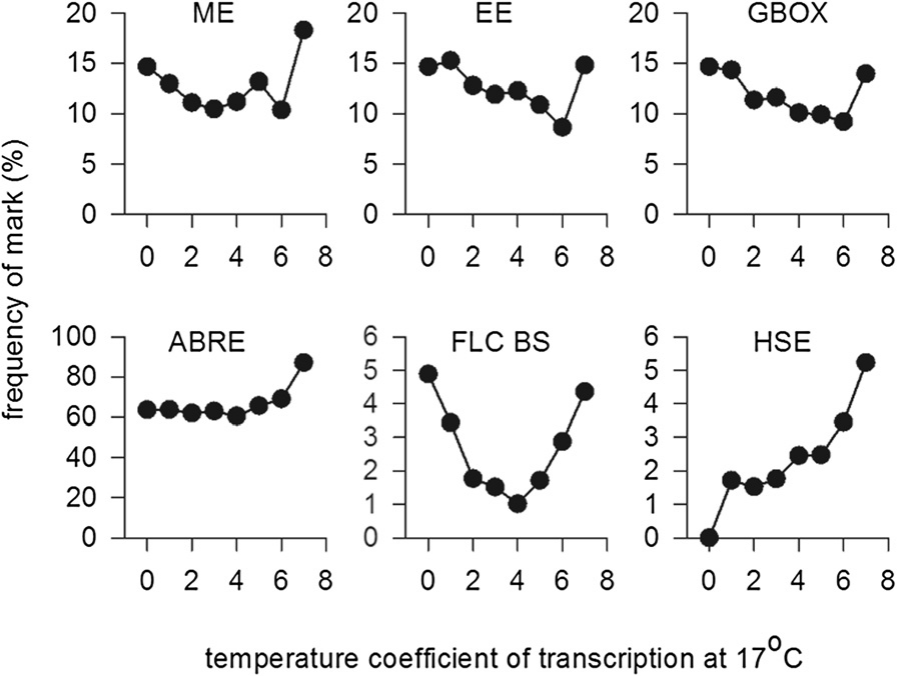
**Transcription factor binding site frequency in 1 kb region upstream of the ATG of all genes in dataset, classified by transcription rate Q**_**10**_**.** Numbers of genes in each Q_10_ group are: 0 < Q_10_ < 1, 41; 1 < Q_10_ < 2, 527; 2 < Q_10_ < 3, 2,039; 3 < Q_10_ < 4, 2,447; 4 < Q_10_ < 5, 1,389; 5 < Q_10_ < 6, 526; 6 < Q_10_ < 7, 174; 7 < Q_10_ < 8, 82. Circadian binding sites were determined using data from Covington *et al*. [19]; FLC binding site data are available from Deng *et al*. [22].

Of the transcription factor binding sites tested, clear associations between Q_10_ and the presence of FLC binding sites and heat shock elements (HSEs) could be detected (Figure 6). As expected, HSEs were more frequent in the promoters of genes with high transcription rate Q_10_s, showing that HSEs confer increased transcription in response to warmer temperatures, even throughout the ambient range. In contrast, FLC binding sites were depleted in genes with average Q_10_s and more frequent in the promoters of genes with Q_10_s below 2 or above 5. This supports an important role for FLC in configuring the ambient temperature response [17], and suggests that FLC can act as a repressor or activator of transcription in different circumstances. Morning elements (MEs), evening elements (EEs), and G-boxes (bound by PIF subfamily transcription factors) showed a similar trend in that their frequency was marginally higher in the promoters of genes with lower Q_10_s, but then increased in the highest Q_10_ category. Interestingly, ABREs were also present at higher frequency in the promoters of gene with the highest Q_10_s. Together this suggests that high transcription rate Q_10_s can be conferred by the combined action of multiple transcription factors.

## Discussion

Measurements of Q_10_ of specific cellular processes are not common: usually these are limited to whole physiology, such as growth or gas exchange [23]. Here we show we can directly measure transcript synthesis and decay rates genome-wide in plants and use these data to calculate the temperature coefficients of synthesis and decay, which have means of 3.6 and 3.3, respectively, at 17°C (Figure 3). Large but balanced passive temperature effects on transcription and decay rate are observed for the majority of genes, and these ensure that mRNA abundance is constant at different growth temperatures. This will necessarily result in increased mRNA turnover at warmer temperatures, which will have a large energy cost to plant cells [24]. Importantly, differential control of transcript levels by ambient temperature is imparted predominantly by temperature-dependent transcriptional effects: these are presumably active processes because they produce changes in overall levels by failing to match passive effects on decay rate by virtue of high or low Q_10_s. Thus, a mRNA whose level increases at warmer temperatures does so because the transcription rate Q_10_ exceeds the passive increases in decay rate.

In order to generate an ambient temperature signal at a locus it is therefore necessary that the thermal response of this locus must deviate from the mean response (Figure 4); however, it follows that such a response must be relatively rare, otherwise it will itself define the mean. Previously it has been suggested that epigenetic effects play a role in the temperature control of transcription rates, with a global role ascribed to H2A.Z and a role for H3K27me3 specifically in the case of *FLC* regulation [2,25]. We found that the presence of less common repressive chromatin states, such as gene body H2A.Z at 22°C, is correlated with reduced transcription rate Q_10_ between 17°C and 27°C (Figure 5). In contrast, more common chromatin states, such as H3K4me3, H3k36me2/3 and cytosine methylation, are biased towards genes with average transcription rate Q_10_s. This is consistent with a view that the thermal responses of common states define the mean response, by virtue of their high frequency and similar properties. The opposite Q_10_ associations of DNA methlyation and H2A.Z is consistent with their known antagonistic roles [26].

Our data do not support a role for ARP6-dependent processes in generating a global transcriptional response to warm ambient temperatures, because genes with high transcription rate Q_10_s are expressed at normal levels in *arp6* mutants (Figure 5C). However, we found a clear association between high gene body H2A.Z at 22°C and low transcription rate Q_10_ between 17°C and 27°C and this relationship is corroborated by the observation that genes with low transcription rate Q_10_s are specifically mis-regulated in *arp6* mutants at 12°C. Because this association specifically involves genes down-regulated in *arp6* mutants at 12°C [2] (Figure 5C), gene body H2A.Z reduces Q_10_ by catalyzing transcription at cooler temperatures, relative to warmer temperatures. Our data are consistent with a model in which chromatin states have distinct activation energies of transcription, and that combining them in specific abundance ratios can produce an ambient temperature response, much like combining two metals into a bimetallic strip can be used to create a thermometer. Alternatively, a 10°C rise in temperature could cause a mass rearrangement of multiple chromatin marks that precipitates Q_10_ variation. In principle, either could be used to generate a biological thermometer, but evidence is required that this process is transduced by plants into a temperature signal, as has been proposed during flowering time control or seed development [3,7]. The chromatin state data used for our analysis were chosen because the analyses were carried out at 22°C, equidistant between 17°C and 27°C. If temperature affects the frequency or location of less common marks, then this may also add an additional layer of complexity.

H3K27me3 presence at 22°C is associated preferentially with extreme Q_10_s across this temperature range (Figure 5A). Further work is necessary to understand whether it has a general role in transducing temperature signals. In the case of *FLC*, H3K27me3 is known to be required to stabilize transcription states but may also have a role in signal transduction [25,27,28].

## Conclusions

Our work reveals that transcription and mRNA decay are subject to both passive and active rate changes by temperature in *A. thaliana*. Our analysis reveals an interaction between chromatin state and transcription rate Q_10_ such that chromatin marks bias transcription rate Q_10_ relative to the mean, in particular gene body H2A.Z, which reduces Q_10_, and H3K27me3, which is associated with extreme temperature responses. These interact with transcription factor activity to confer the observed ambient temperature response of the *Arabidopsis* transcriptome. Our approach shows that measurements of real rates have clear advantages over inferring rate changes from steady state levels, and will be useful for investigating dynamic plant responses to other signals.

## Materials and methods

### Plant growth and labeling

*Arabidopsis* plants, ecotype Columbia, were grown in cycles of 12 hours 80 μmol white light, 12 hours dark. Eleven-day-old seedlings were removed from MS (Murashige and Skoog, Melfords, Ipswich, Suffolk, UK) agar plates and allowed to acclimatize overnight to floating in liquid MS media. They were then labeled by adding 4SU (Carbosynth Compton, Berkshire, UK) to the liquid MS media 10 minutes after dawn. The concentrations added were different for different labeling times and temperatures and optimal concentrations for each set of conditions were determined experimentally using dot blots (see below for method) visualized using streptavidin-HRP (Genscript Piscataway, NJ, USA) to check the levels of 4SU in the RNA. Plant samples were removed from the liquid medium and flash frozen in liquid nitrogen.

### RNA extraction and separation

RNA was extracted from the plant samples using the RNeasy Plant (QIAGEN Germantown, MD, USA). The extracted labeled RNA was then biotinylated and separated on streptavidin columns as previously described [8]. The separated pre-existing RNA was tested by dotting onto Zeta probe membranes (Biorad Hercules, CA, USA) and detecting any remaining biotinylated RNA using streptavidin-HRP (Genscript), ECL plus reagents (GE Life Sciences, Little Chalfont, Buckinghamshire, UK) and a light detection system. RNA samples were checked for integrity using Agilent Bioanalyzer pico or nanochips, according to the manufacturer’s protocols.

### Microarray analysis

The RNA samples were labeled and hybridized to Affymetrix ATH1 chips using the manufacturer's protocols: these included three biological replicates for each sample type (total at end of labeling, labeled newly transcribed, unlabeled pre-existing and total at start of labeling) for each temperature (17°C and 27°C). Arrays were processed using Mas5 with detection calls. Genes declared absent in one or more of the 12 total RNA samples were not included in further analysis. Transcript levels were scored as being significantly up- or down-regulated with temperature using SAM [29] with a false discovery rate of 1%.

### Normalization of newly transcribed and pre-existing RNA by linear regression

Because equal amounts of RNA for the newly synthesized (NS), pre-existing (PE) and total (T) samples were loaded on the chips, measured transcript levels in the PE and NS samples were scaled to be directly comparable to the transcript level measured in the T dataset. To achieve this the ratios of the T mRNA accounted for by the NS and PE RNA pools were calculated by linear regression as described [8] based on the fact that for each gene the sum of the expression value ratios PE/T and = NS/T must equal 1 (100% of the total). But because equal amounts of RNA were loaded on the chips it is necessary to use correction factors to get back to this value of 1.

Correction factors that enable the transcript level of each gene in the NS and PE samples to be scaled to be directly comparable to those in the T sample were calculated assuming:

(1) $$\mathrm{Cp}.\left(\mathrm{PE}/T\right)+\mathrm{Cn}.\left(\mathrm{NS}/T\right)=1,$$

where Cp is the correction factor for pre-existing RNA and Cn is the correction factor for newly transcribed RNA. This rearranges to give:

(2) $$\mathrm{PE}/T=\left(1/\mathrm{Cp}\right)-\left(\mathrm{NS}/T\right).\left(\mathrm{Cn}/\mathrm{Cp}\right)$$

To calculate the correction factors, NS/T for each gene is plotted against PE/T for each gene, giving a scatter plot and linear regression (Figure S5 in Additional file 1). The correction factors were calculated from the linear regression equation where 1/Cp is the intercept and Cn/Cp is the slope.

### Calculation of half-lives

Half-lives were calculated assuming that mRNA decay follows a first order exponential decay kinetic [30,31]. For each gene, we can use the equation:

(3) $$t1/2=-\mathrm{tL}\;.\;\ln\left(2\right)/\ln\left(P/A_{0}\right)$$

where t1/2 is the half-life in hours, tL is the labeling time in hours, P is the scaled amount of pre-existing transcript remaining at the end of the labeling time and A_0_ is the amount of transcript at the start of the labeling time.

### Calculation of transcription rate

The amount of RNA transcribed for each gene during the labeling period is equal to the amount of RNA detected at the end of the labeling plus the amount of NS RNA to have decayed during the labeling period. To get the amount synthesized in one hour we can use the formula:

(4) $$\frac{\mathrm{N\lambda}}{1-\left(e^{\left(-\lambda \;\mathrm{tL}\right)}\right)}$$

where N is the scaled amount of newly transcribed RNA remaining at the end of the labeling time, tL is again the labeling time, and the decay constant λ is:

$$\mathrm{Ln}\left(2\right)/t1/2.$$

### Transcript feature analysis

cDNA and UTR length, GC content, U content and intron number as well as the presence or absence of short, potentially regulatory, sequences were extracted from the latest sequence files on the TAIR website [32] using Perl scripts (available on request).

### Real-time RT-PCR

RNA was labeled, extracted, separated and checked as above. First-strand cDNA was synthesized with 500 ng (or 100 ng for newly transcribed samples) of total RNA in 20 μl reactions, Superscript II Reverse Transcriptase (Invitrogen Carlsbad, CA, USA) and oligo dT (Invitrogen), according to the manufacturer’s protocol. Water (180 μl) was added and 2.5 μl of this was used in the quantitative PCR step. Primers are listed in Table S2 in Additional file 1. Quantitative PCR was carried out on the same samples for a number of genes and these were normalized collectively in a similar fashion to the microarrays, with the levels for the un-separated samples normalized to both *UBQ10* and *TUB2*.

### Data availability

Microarray data generated for this manuscript are available at the Gene Expression Omnibus under accession number GSE53071. Further datasets used in this analysis were GSE39045 [20] and GSE28398 [21].

## Abbreviations

4SU: 4-thiouracil; ABRE: ABA response element; CG: cool gene; GO: Gene Ontology; HSE: heat shock element; NS: newly synthesized; PCR: polymerase chain reaction; PE: pre-existing; T: total; TAIR: The Arabidopsis Information Resource; UG: unchanged gene; UTR: untranslated region; WG: warm gene.

## Competing interests

The authors declare that they have no competing interests.

## Authors’ contributions

KS-L designed experiments, carried out experiments, analyzed data and wrote the paper. MJC, BF and DR advised on mathematical and statistical aspects of the analysis. SP designed experiments and wrote the paper. All authors read and approved the final manuscript.

## Supplementary Material

Additional file 1 **Plants take up 4SU and incorporate it into RNA. Figure S2.** 4SU is not toxic to plants and incorporation rate is temperature-dependent. **Figure S3.** Scatter plots used in linear regression analysis. **Figure S4.** Quantitative RT-PCR independently confirms rates calculated from microarray data using an extended time-series analysis. **Figure S5.** Scatter plots showing a lack of correlation between transcription and decay rates at both 27°C and 17°C and various transcript features: **(a,b)** 5′ UTR length; **(c,d)** 3′ UTR length; **(e,f)** cDNA length; **(g,h)** uracil content; **(i,j)** GC content; **(k,l)** number of introns. **Figure S6.** No effect of intron number on synthesis and decay rate Q_10_ distribution in *Arabidopsis*. **Table S2.** List of quantitative PCR primers used for rate verification analysis shown in Figure S2 in Additional file 1. Click here for file

Additional file 2: Table S1 Genome-wide data for mRNA synthesis and decay rates and temperature coefficient. Click here for file
