# Supplementary material for: Direct measurement of transcription rates reveals multiple mechanisms for configuration of the Arabidopsis ambient temperature response
Source: Genome Biol. 2014 Mar 3;15(3):R45. doi: 10.1186/gb-2014-15-3-r45 (PMC4053849; doi:10.1186/gb-2014-15-3-r45)
Supplement: Additional file 1 — Plants take up 4SU and incorporate it into RNA. Figure S2. 4SU is not toxic to plants and incorporation rate is temperature-dependent. Figure S3. Scatter plots used in linear regression analysis. Figure S4. Quantitative RT-PCR independently confirms rates calculated from microarray data using an extended time-series analysis. Figure S5. Scatter plots showing a lack of correlation between transcription and decay rates at both 27°C and 17°C and various transcript features: (a,b) 5′ UTR length; (c,d) 3′ UTR length; (e,f) cDNA length; (g,h) uracil content; (i,j) GC content; (k,l) number of introns. Figure S6. No effect of intron number on synthesis and decay rate Q10 distribution in Arabidopsis. Table S2. List of quantitative PCR primers used for rate verification analysis shown in Figure S2 in Additional file 1. [file gb-2014-15-3-r45-S1.pdf]

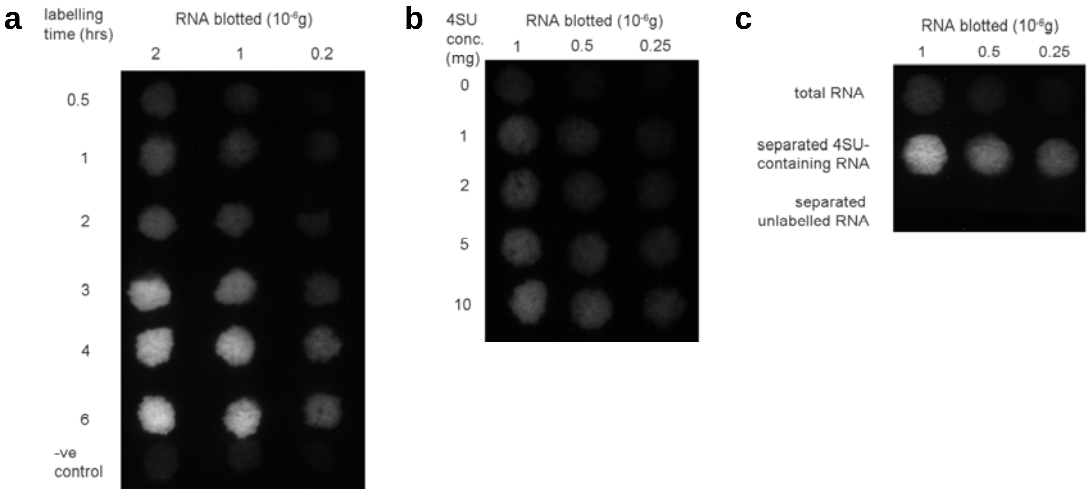

Supplementary Figure 1. Plants take up 4SU and incorporate into RNA. **a**. A labelling timecourse using whole seedlings floated on MS medium containing 1.5mM 4SU at 27°C, determined by biotin labelling, dot blotting and detection by streptavidin-HRP (see methods for details). Signal above background (no 4SU -ve control) is obvious after 30 minutes. **b**. Experiment to optimise the concentration of applied 4SU after 1 hour of labelling. 1.5mM was chosen because high labelling can reduce the number of transcripts binding to the column and lower recovery. **c**. Plant RNA extractions can be biotinylated and separated into 4SU-labelled and unlabelled fractions. RNA was collected after 1 hour floatation on 1.5mM 4SU at 27°C.

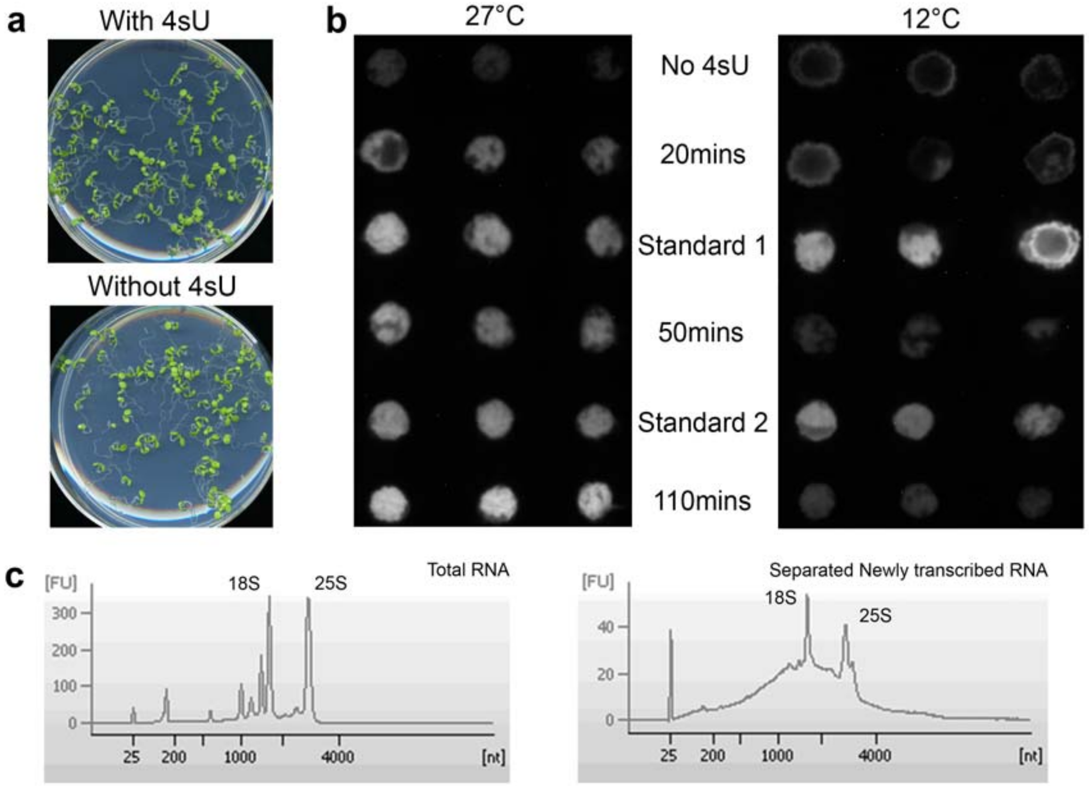

Supplementary Figure 2. 4SU is not toxic to plants and incorporation rate is temperature-dependent. a. 7 day old plants grown either with or without 1.5mM 4sU (4-thiouridine) **b**. Dot blots showing total RNA samples from plants grown at two different temperatures (27°C and 12°C), treated with 4sU for the times shown. The brightness of the dot corresponds to the level of labelled RNA in the sample as biotin bound to the label is detected using streptavidin-HRP and ECL reagents. The standards were identical on both blots to enable comparison. **c**. Electropherograms of Total RNA and separated newly transcribed RNA. The rRNA peaks are labelled. Relative to the amount of other RNA present, the rRNA is depleted in the newly transcribed sample. Charts show RNA collected after 2 hour labelling at 17°C and separated for microarray analysis.

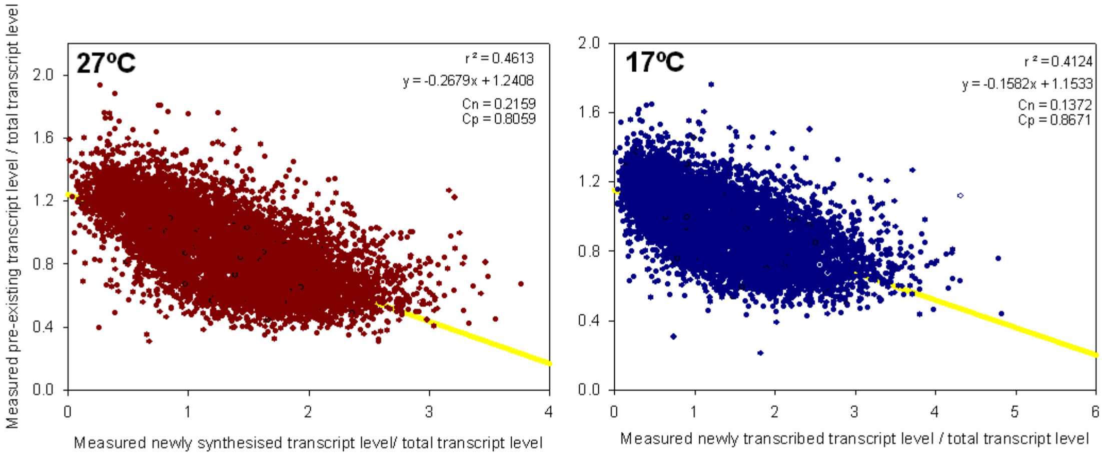

Supplementary Figure 3. Scatter plots used in linear regression analysis. Ratios of measured newly transcribed to total transcript plotted against ratios of pre-existing to total transcript (see methods for details). The line of best fit were used to calculate the correction factors (Cn and Cp) for each temperature, needed to scale the expression level of each gene in the newly transcribed an pre-existing samples to the level in the total sample.

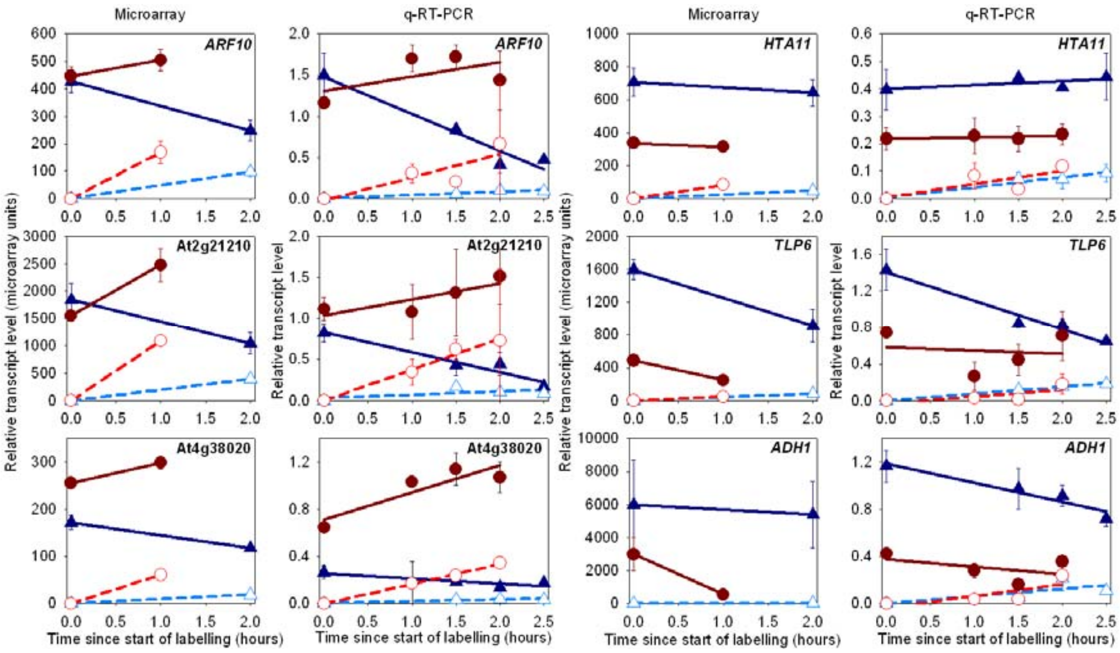

Supplementary Figure 4. qRT-PCR independently confirms rates calculated from microarray data using an extended time-series analysis. Genes were chosen which exhibited either a higher transcript abundance at 27°C or at 17°C, showing array data with q-RT-PCR data in a side-by-side comparison. In each case datapoints are the mean and standard error of three biological replicates per time-point. Newly transcribed transcript levels are calculated using linear regression analysis (Dolken et al., 2008; see methods). Solid lines with closed symbols represent total transcript abundance, dashed lines with open symbols represent calculated newly transcribed RNA. Blue lines indicate RNA levels at 17°C, red at 27°C.

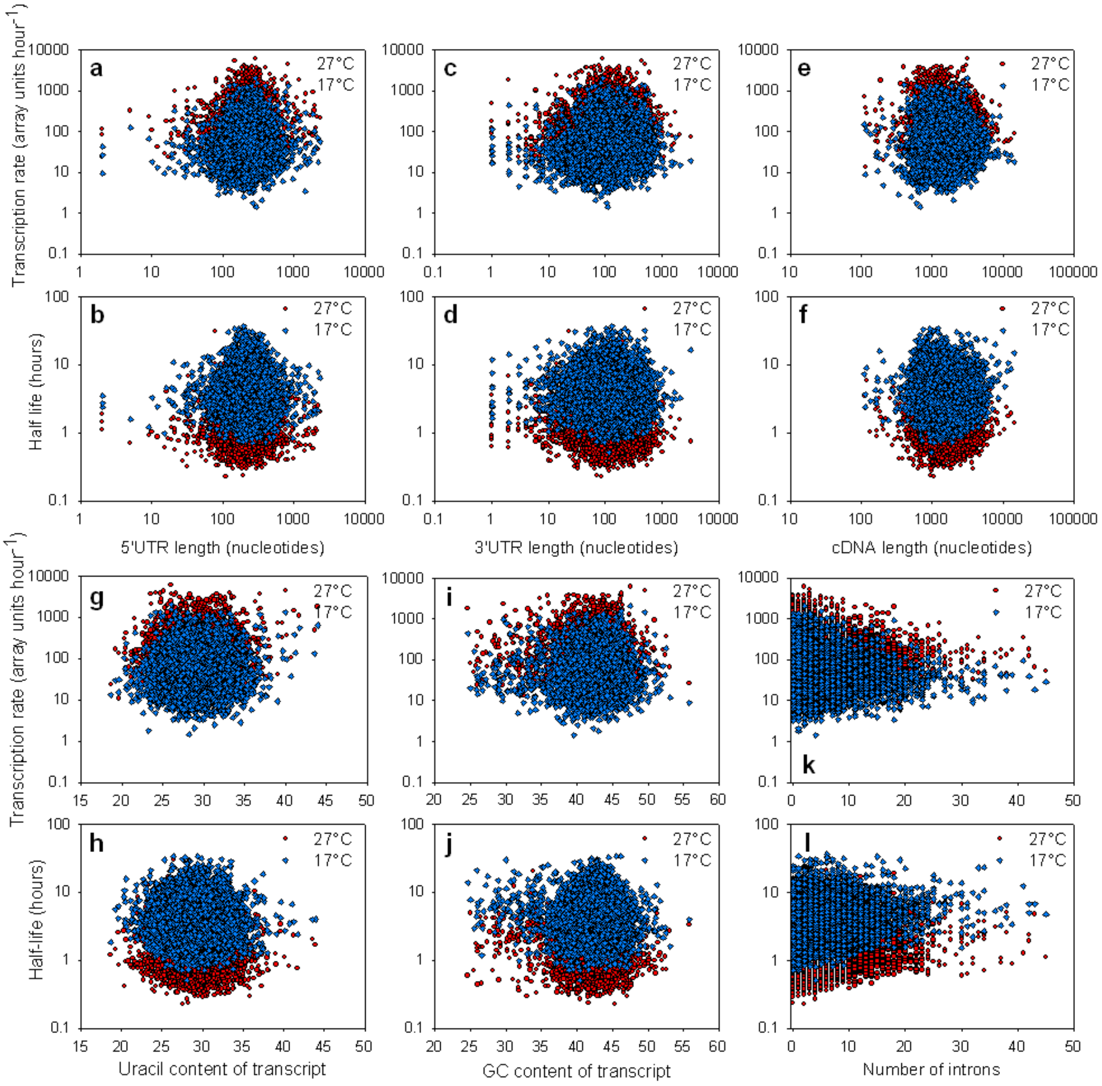

Supplementary Figure 5. Scatter plots showing a lack of correlation between transcription and decay rates at both 27 and 17°C and various transcript features: **a** and **b**. 5'UTR length; **c** and **d**. 3'UTR length; **e** and **f**. cDNA length; **g** and **h**. Uracil content; **i** and **j**. GC content; **k** and **l**. number of introns.

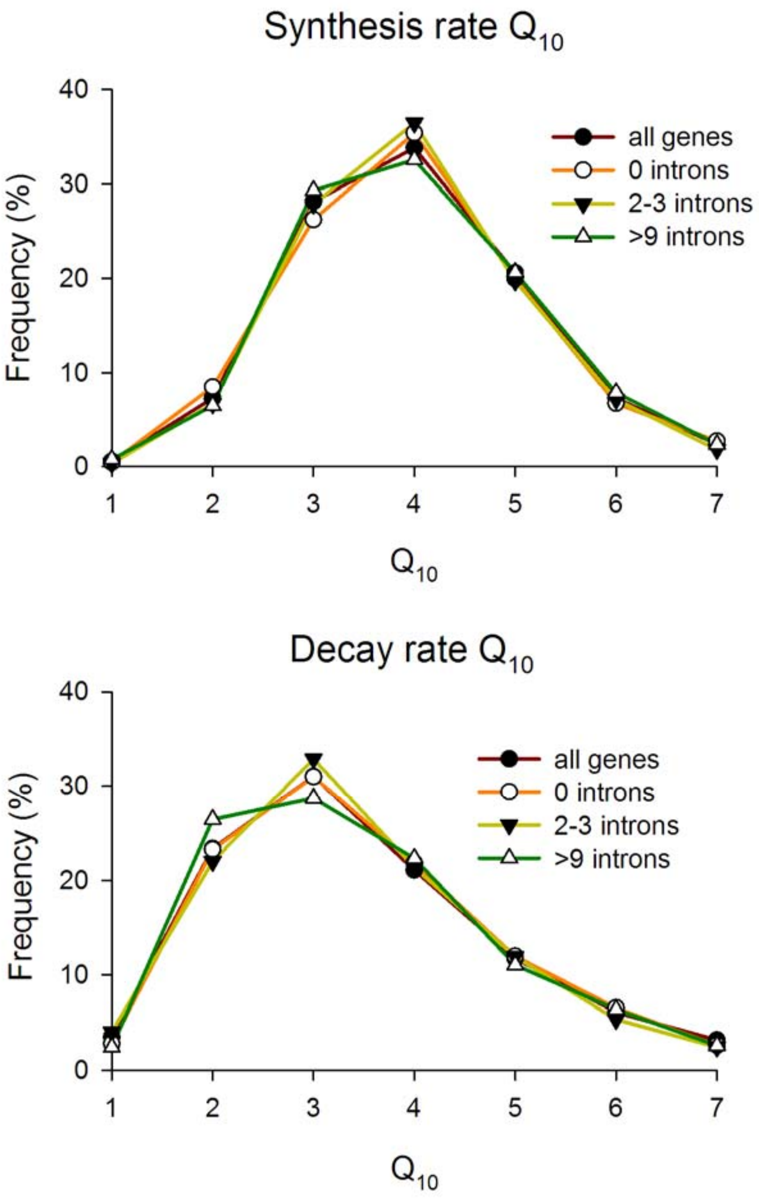

Supplementary Figure 6. No effect of intron number on synthesis and decay rate Q_{10} distribution in Arabidopsis. Q10 distributions were determined for genes without introns (n=610), with a low intron number (2 or 3; n= 1064) and with a high intron number (≥10; n=1111) and compared to the whole dataset (n-7291).

Supplementary Table 2. List of qPCR primers used for rate verification analysis shown in Supplementary Figure 2.

RT_At2g21210_F	GCAGAAGAAGAGTTTGGGTTT
RT_At2g21210_R	TGAAGTGCTAAGGCGAGAGG
RT_At3g23550_F	CCTCTGTGATGTTCGCTTCTC
RT_ At3g23550_R	GGTGACGGTAGCCCAAGA
RT_TLP6_F	TCATTGCGGTTACTGTCGTC
RT_TLP6_R	ACCAACACCAACACCTCTCC
RT_ AT1G19430_F	GGATTTGGGTGAAGATGACG
RT_ AT1G19430_R	AGGACCAGAAGAAGACGAAGC
RT_AT4G38020_F	TCATGGCTCTGTTCTTGTCG
RT_AT4G38020_R	TGGTCATTCCTTGCTTATTCG
RT_ARF10_F	ATTACGCTTTTGCCACTTCC
RT_ARF10_R	CCGTTACCGTCAGAAGAAGG
RT_HTA11_F	CAAAGGTGGAAAAGGACTCG
RT_HTA11_R	CAGAGCGAGAGATGGGTTTC
RT_UBQ10_F[6]	CACACTCCACTTGGTCTTGCGT
RT_UBQ10_R[6]	TGGTCTTTCCGGTGAGAGAGTC
RT_TUB_F [22]	TGGCATCAACTTTCATTGGA
RT_TUB_R [22]	ATGTTGCTCTCCGCTTCTGT
